# Correlates of Protection against Norovirus Infection and Disease—Where Are We Now, Where Do We Go?

**DOI:** 10.1371/journal.ppat.1005334

**Published:** 2016-04-26

**Authors:** Sasirekha Ramani, Mary K. Estes, Robert L. Atmar

**Affiliations:** 1 Department of Molecular Virology and Microbiology, Baylor College of Medicine, Houston, Texas, United States of America; 2 Department of Medicine, Baylor College of Medicine, Houston, Texas, United States of America; University of Kentucky, UNITED STATES

Human noroviruses (NoV) are the leading cause of foodborne gastroenteritis and endemic diarrheal disease across all ages in the United States, cause nearly 50% of all gastroenteritis outbreaks worldwide, and are rapidly replacing rotavirus as the predominant gastrointestinal pathogen in pediatric populations [1]. Modelling studies show that vaccination can offer significant healthcare and economic benefits, depending on vaccine cost, protective efficacy, and protection duration [2]. We stand at the threshold of exciting progress in NoV vaccine evaluation, with several vaccine candidates in development. Furthest along are virus-like particles (VLPs), composed of the NoV major capsid protein and produced in a recombinant baculovirus expression system. VLP vaccines are safe, immunogenic, and were efficacious in a proof-of-principle human experimental infection study [3]. As development transitions from small preclinical and clinical studies to larger clinical trials—and eventually to applications for licensure—it is important to review the current knowledge on correlates of protection (CoPs) against NoV infection and disease (Table 1), as well as to identify and address the remaining challenges (Table 2). This is critical to understand the basis of vaccine efficacy and to provide direction for the design and implementation of future NoV vaccine studies.

**Table 1:** Correlates of protection.

Study Design	Correlate of protection	Time	Outcome	Reference
Human	Host genetics	Preexposure	Infection and illness	[5,7] (and other studies)
Experimental	Serum histoblood group antigen (HBGA)-blocking antibody	Preexposure	Infection and illness	[10]
Challenge	Serum hemagglutination inhibition antibody	Preexposure	Illness	[23]
	Salivary Immunoglobulin A (IgA)	Preexposure	Illness	[14]
		Postexposure (rapid response)	Infection	[7]
	Fecal IgA	Preexposure	Peak virus shedding	[14]
		Postexposure (day 7)	Duration of virus shedding	[14]
	Virus-specific memory Immunoglobulin G (IgG) cells	Preexposure	Illness	[14]
Vaccine	Serum histoblood group antigen (HBGA)-blocking antibody	Prechallenge	Infection and illness	[3]
Studies	Serum IgA	Prechallenge	Infection and illness	[11]

**Table 2:** Questions for future NoV vaccine studies.

Key Questions	Implications
What is the relative importance of the different immune CoP?	• Provide insight into mechanism(s) of protective immunity to NoV
	• Influence methods of endpoint assessments in future vaccine studies
	• Define choice of adjuvants and route of immunization in vaccine studies
Is immune response to NoV heterotypic?	• Influence the number of NoV targets to be included in vaccine formulations
	• Indicate if vaccines will need to be updated frequently
What is the duration of protective immunity to NoV?	• Define frequency of vaccination
	• Utility at broad (population) level versus specific settings (e.g., cruise ship travelers, military personnel, nursing homes, etc.)
What is the influence of age at vaccination on immune response?	• Define the applicability of NoV vaccines in extremes of age among the groups with greatest disease burden
Is immunological priming required for robust immunity?	• Influence the applicability of NoV vaccines to pediatric populations

## How Do Host Genetics Influence Protection from NoV Infection?

Human experimental challenge studies conducted in the 1970s identified individuals resistant to infection with the prototype strain, Norwalk virus (NV, genotype GI.1); the protective effect could not be attributed to the presence of serum antibodies [4]. Evidence for the involvement of host genetics came in the early 2000s, when an association between an individual’s ABO histoblood group antigen (HBGA) type and risk of NV infection was identified [5]. HBGAs are blood group antigens present on epithelial cell surfaces and in mucosal secretions, and the expression profile is based on an individual’s ABH secretor and Lewis genotypes. HBGAs are cell attachment factors for NoVs [6]. Resistance to NV is mediated by the absence of a functional fucosyl transferase 2 (FUT2) gene (secretor-negative genotype) [7]. The association between HBGA expression and susceptibility to specific NoV genotypes is now well established [1]. Apart from isolated cases, functional FUT2 gene expression is required for infection with most variants of the predominant NoV genotype, GII.4. This genotype binds a diverse range of HBGAs compared to other genotypes, possibly explaining its predominance. The epidemiology of NoV is complex, with more than 30 genotypes known to cause infections in humans [8]. Since HBGA specificity varies among different NoV genotypes, it is likely that most persons will be susceptible to at least some genotypes [9].

## What Immune Effectors Are CoP against NoV Infection and Disease?

Current knowledge on immune responses to human NoVs comes predominantly from experimental human infection models and clinical studies with VLP vaccines. The first immune CoP against NoV gastroenteritis was identified in a NV human volunteer challenge study, in which prechallenge serum levels of functional antibodies that block binding of NV VLPs to HBGAs correlated with lower risk of illness [10]. HBGA-blocking antibodies were also a CoP against NoV infection and disease following vaccination with monovalent (GI.1) and bivalent (GI.1 and GII.4) VLP vaccine formulations [3,11]. However, following intramuscular immunization with the bivalent vaccine and challenge with an infectious virus, a discordance was observed between the levels of HBGA-blocking antibodies required to achieve protection in vaccinees and placebo recipients [11]. Infectious virus challenge in placebo subjects showed that high levels of HBGA-blocking antibodies were associated with a lower frequency of infection and illness. However, the same pattern did not hold true in the vaccine recipients. In this group, the prechallenge levels of HBGA-blocking antibodies were significantly higher than those in the placebo recipients, but these higher titers were not necessarily associated with reduction in infection or illness. In the vaccinated group, the levels of HBGA-blocking antibodies required to achieve protection was greater than the placebo recipients. The overall immune response to the bivalent vaccine in the challenge study was similar to a previous study assessing immunogenicity of this formulation [12]. This finding mimics results seen with parenteral, inactivated influenza virus vaccines [13] and is a potential barrier for the broad use of HBGA-blocking antibody titers as CoP in NoV vaccine studies. Mucosal and cellular immunity also appear to be important in protection against NoV disease. In NV experimental challenge studies, prechallenge levels of virus-specific salivary IgA and IgG memory B cells are a CoP against illness [14]. Prechallenge, NV-specific salivary IgA levels also correlate with reduced severity of gastroenteritis. Rapid salivary IgA response following NV challenge was previously demonstrated to be protective against infection [7]. Prechallenge, NV-specific fecal IgA levels are associated with a lower peak viral load, while levels on day seven postinfection correlate with a shorter duration of virus shedding [14]. The CoPs against NoV infection and disease identified thus far are summarized in Table 1.

## Identification of Multiple Immune CoP: One Too Many to Test?

While several immune CoP have been identified, none were an absolute CoP. Some are protective against disease, while others are protective against both infection and disease. Most CoPs were identified from a single human challenge study involving a small number of participants. It is likely that at least some CoP covary, making it difficult to identify the best predictor of protection. This raises fundamental questions about the mechanisms of protective immunity against NoV. Analyzing the relative contribution of each CoP will be important in order to make decisions on immunization route and adjuvant use and is likely to add complexity to vaccine study designs. It is also possible that studies on little-explored aspects of NoV immunity, such as T cell and cytokine responses, will lead to new discoveries. In persistent murine NoV infections, mice treated with exogenous interferon lambda (IFN-λ) showed viral clearance, suggesting a role for innate immunity [15]. Antibiotic treatment of mice increased this effect, suggesting a role for microbiome-mediated alterations in innate immune response [16]. In a recent study, the presence of HBGA-expressing enteric bacteria was found to be important for infection of B cells with human and mouse noroviruses [17]. While murine NoV infections are clinically different from human NoV disease, these studies suggest the possibility of additional effectors as CoP that could influence vaccine evaluation.

## What Is the Duration of Protective Immunity?

Early human experimental infection studies suggested that protective immunity to NoV is short-lived and does not extend to strains beyond the challenge virus. While these results may possibly be due to high inoculum dosages, they pose important questions to vaccine implementation. The duration of protection has not been assessed beyond six to 12 months in any recent study. In a mathematical model, immunity following natural NoV infection was estimated to last four to nine years [18], but this remains to be validated in field efficacy studies. Characterizing the duration of persistence of different CoPs can identify the mediators of short-term and long-term protection. Vaccines inducing long-term protection are likely to have broad, population-level applications, while the induction of short-term protection may be valuable in specific settings.

## Are There Epidemiological Challenges to Broadly Protective Immunity?

NoVs evolve rapidly by antigenic drift and recombination, resulting in a complex epidemiology. NoV disease in humans is caused by more than 30 genotypes in three genogroups, and this genetic diversity poses a potential problem in developing vaccination strategies to prevent infection and illness. While most infections are caused by the GII.4 genotype, new variants emerge every two to three years, replacing the previously dominant variant [1]. The epochal evolution of strains and changes in HBGA-binding patterns pose important challenges to the development of broadly effective vaccines. The lack of cross-protection and short-term protection observed in early studies suggests a need for frequent vaccination as well as the inclusion of multiple genotypes in a vaccine. In this context, perhaps the most exciting finding from recent challenge studies and vaccine trials is that immune responses to NoVs appear to be heterotypic. Following NV infection or immunization with the GI.1 and GII.4 bivalent vaccine, HBGA-blocking antibodies were induced to heterotypic GI and GII strains, including to GII.4 variants that were not in circulation at the time of infection or sample collection [19,20]. Given that HBGA-blocking antibodies are a CoP for noroviruses, these data suggest that vaccination could induce broadly cross-reactive blocking antibodies, and thus protection could extend to genotypes apart from the ones included in the vaccine formulation. Although the percentage of responders and the magnitude of increase in heterotypic antibody titers were modest compared to the homologous response, these results hold promise for the effectiveness of simpler vaccines rather than multivalent formulations containing many genotypes. This remains to be tested in field efficacy studies. How waning levels of protective antibodies influence the emergence of new variants is an important question to address as the induction of broadly cross-reactive antibodies contrasts with reports that immune, pressure-mediated antigenic variation in epitopes surrounding the HBGA-binding domain of the capsid protein drives the emergence of new variants [21].

## Will Clinical Trial Efficacy Results Translate to Populations at Greatest Risk?

Human challenge studies and clinical trials with VLP vaccines have largely been conducted in genetically susceptible, healthy adults in well-controlled environments. Following vaccination, individuals were challenged with NoV genotypes included in the vaccine. These conditions are different from field settings, where multiple NoV genotypes and variants co-circulate among individuals with different levels of genetic susceptibility to NoV infection. This is particularly relevant for populations at greatest risk of illness: infants less than five years of age, in whom a high burden of disease has been demonstrated, and the elderly, in whom NoV infections result in high morbidity and some mortality [1,22]. How the present findings will translate to these populations remains to be elucidated, as most data are from healthy adults with preexisting NoV-specific antibodies. The need for immunological priming for development of a robust, broadly reactive immune response could have significant implications for vaccine efficacy in young children. While priming may not be an issue for the elderly, protective responses could be adversely affected by diminished immune responses in elders and those with other conditions affecting immunity. Field efficacy studies are therefore required to obtain an accurate estimate of protection in the natural environment.

## Summary and Conclusions

Coordinated efforts of epidemiological studies and surveillance networks are establishing the significant public health impact of NoVs as a gastrointestinal pathogen across different populations and age groups. This defining phase provides a platform to focus on pertinent questions and challenges for future NoV vaccine studies. As promising vaccine candidates move into field trials, corresponding studies to assess the relative importance of the different CoPs will help identify the best correlates and lead to improved prevention strategies.

## References

[1] Ramani S, Atmar RL, Estes MK. Epidemiology of human noroviruses and updates on vaccine development. Current opinion in gastroenterology. 2014;30(1):25–33. Epub 2013/11/16. 10.1097/MOG.0000000000000022 24232370 PMC3955997

[2] Bartsch SM, Lopman BA, Hall AJ, Parashar UD, Lee BY. The potential economic value of a human norovirus vaccine for the United States. Vaccine. 2012;30(49):7097–104. Epub 2012/10/03. 10.1016/j.vaccine.2012.09.040 23026689 PMC3517973

[3] Atmar RL, Bernstein DI, Harro CD, Al-Ibrahim MS, Chen WH, Ferreira J, et al Norovirus vaccine against experimental human Norwalk Virus illness. The New England journal of medicine. 2011;365(23):2178–87. Epub 2011/12/14. 10.1056/NEJMoa1101245 22150036 PMC3761795

[4] Parrino TA, Schreiber DS, Trier JS, Kapikian AZ, Blacklow NR. Clinical immunity in acute gastroenteritis caused by Norwalk agent. The New England journal of medicine. 1977;297(2):86–9. Epub 1977/07/14. 10.1056/NEJM197707142970204 . 405590

[5] Hutson AM, Atmar RL, Graham DY, Estes MK. Norwalk virus infection and disease is associated with ABO histo-blood group type. The Journal of infectious diseases. 2002;185(9):1335–7. 10.1086/339883 . 12001052

[6] Marionneau S, Ruvoen N, Le Moullac-Vaidye B, Clement M, Cailleau-Thomas A, Ruiz-Palacois G, et al Norwalk virus binds to histo-blood group antigens present on gastroduodenal epithelial cells of secretor individuals. Gastroenterology. 2002;122(7):1967–77. . 12055602 10.1053/gast.2002.33661 PMC7172544

[7] Lindesmith L, Moe C, Marionneau S, Ruvoen N, Jiang X, Lindblad L, et al Human susceptibility and resistance to Norwalk virus infection. Nature medicine. 2003;9(5):548–53. Epub 2003/04/15. 10.1038/nm860 . 12692541

[8] Vinje J. Advances in laboratory methods for detection and typing of norovirus. J Clin Microbiol. 2015;53(2):373–81. 10.1128/JCM.01535-14 24989606 PMC4298492

[9] Huang P, Farkas T, Zhong W, Tan M, Thornton S, Morrow AL, et al Norovirus and histo-blood group antigens: demonstration of a wide spectrum of strain specificities and classification of two major binding groups among multiple binding patterns. Journal of virology. 2005;79(11):6714–22. 15890909 10.1128/JVI.79.11.6714-6722.2005 PMC1112114

[10] Reeck A, Kavanagh O, Estes MK, Opekun AR, Gilger MA, Graham DY, et al Serological correlate of protection against norovirus-induced gastroenteritis. The Journal of infectious diseases. 2010;202(8):1212–8. Epub 2010/09/08. 10.1086/656364 20815703 PMC2945238

[11] Atmar RL, Bernstein DI, Lyon GM, Treanor JJ, Al-Ibrahim MS, Graham DY, et al Serological Correlates of Protection against a GII.4 Norovirus. Clinical and vaccine immunology: CVI. 2015;22(8):923–9. 10.1128/CVI.00196-15 . 26041041 PMC4519714

[12] Treanor JJ, Atmar RL, Frey SE, Gormley R, Chen WH, Ferreira J, et al A Novel Intramuscular Bivalent Norovirus Virus-Like Particle Vaccine Candidate-Reactogenicity, Safety, and Immunogenicity in a Phase 1 Trial in Healthy Adults. The Journal of infectious diseases. 2014; 210(11):1763–7. Epub 2014/06/22. 10.1093/infdis/jiu337 . 24951828 PMC8483568

[13] Ohmit SE, Petrie JG, Cross RT, Johnson E, Monto AS. Influenza hemagglutination-inhibition antibody titer as a correlate of vaccine-induced protection. The Journal of infectious diseases. 2011;204(12):1879–85. 10.1093/infdis/jir661 . 21998477

[14] Ramani S, Neill FH, Opekun AR, Gilger MA, Graham DY, Estes MK, et al Mucosal and Cellular Immune Responses to Norwalk Virus. The Journal of infectious diseases. 2015; 212(3):397–405. Epub 2015/01/31. 10.1093/infdis/jiv053 . 25635121 PMC4817641

[15] Nice TJ, Baldridge MT, McCune BT, Norman JM, Lazear HM, Artyomov M, et al Interferon-lambda cures persistent murine norovirus infection in the absence of adaptive immunity. Science. 2015;347(6219):269–73. 10.1126/science.1258100 25431489 PMC4398891

[16] Baldridge MT, Nice TJ, McCune BT, Yokoyama CC, Kambal A, Wheadon M, et al Commensal microbes and interferon-lambda determine persistence of enteric murine norovirus infection. Science. 2015;347(6219):266–9. 10.1126/science.1258025 25431490 PMC4409937

[17] Jones MK, Watanabe M, Zhu S, Graves CL, Keyes LR, Grau KR, et al Enteric bacteria promote human and mouse norovirus infection of B cells. Science. 2014;346(6210):755–9. 10.1126/science.1257147 25378626 PMC4401463

[18] Simmons K, Gambhir M, Leon J, Lopman B. Duration of immunity to norovirus gastroenteritis. Emerging infectious diseases. 2013;19(8):1260–7. Epub 2013/07/24. 10.3201/eid1908.130472 23876612 PMC3739512

[19] Czako R, Atmar RL, Opekun AR, Gilger MA, Graham DY, Estes MK. Experimental human infection with Norwalk virus elicits a surrogate neutralizing antibody response with cross-genogroup activity. Clinical and vaccine immunology: CVI. 2015;22(2):221–8. Epub 2014/12/30. 10.1128/CVI.00516-14 25540269 PMC4308873

[20] Lindesmith LC, Ferris MT, Mullan CW, Ferreira J, Debbink K, Swanstrom J, et al Broad Blockade Antibody Responses in Human Volunteers after Immunization with a Multivalent Norovirus VLP Candidate Vaccine: Immunological Analyses from a Phase I Clinical Trial. PLoS Med. 2015;12(3):e1001807. 25803642 10.1371/journal.pmed.1001807 PMC4371888

[21] Lindesmith LC, Beltramello M, Donaldson EF, Corti D, Swanstrom J, Debbink K, et al Immunogenetic mechanisms driving norovirus GII.4 antigenic variation. PLoS Pathog. 2012;8(5):e1002705 10.1371/journal.ppat.1002705 22615565 PMC3355092

[22] Trivedi TK, Desai R, Hall AJ, Patel M, Parashar UD, Lopman BA. Clinical characteristics of norovirus-associated deaths: a systematic literature review. American journal of infection control. 2013;41(7):654–7. Epub 2012/12/26. 10.1016/j.ajic.2012.08.002 . 23266383

[23] Czako R, Atmar RL, Opekun AR, Gilger MA, Graham DY, Estes MK. Serum hemagglutination inhibition activity correlates with protection from gastroenteritis in persons infected with Norwalk virus. Clinical and vaccine immunology: CVI. 2012;19(2):284–7. Epub 2011/12/23. 10.1128/CVI.05592-11 22190401 PMC3272916

